# Effect of Steam to Carbon Dioxide Ratio on the Performance of a Solid Oxide Cell for H_2_O/CO_2_ Co-Electrolysis

**DOI:** 10.3390/nano13020299

**Published:** 2023-01-11

**Authors:** Naouma Bimpiri, Argyro Konstantinidou, Dimitrios Tsiplakides, Stella Balomenou, Kalliopi Maria Papazisi

**Affiliations:** 1 Department of Chemistry, Aristotle University of Thessaloniki, 54124 Thessaloniki, Greece; 2 Chemical Process and Energy Resources Institute, Centre for Research and Technology Hellas, 57001 Thessaloniki, Greece

**Keywords:** co-electrolysis, perovskite oxide, doping, lanthanum chromite, LSCF, solid oxide, SOEC, syngas, steam to carbon dioxide ratio

## Abstract

The mixture of H_2_ and CO, the so-called syngas, is the value-added product of H_2_O and CO_2_ co-electrolysis and the feedstock for the production of value-added chemicals (mainly through Fischer-Tropsch). The H_2_/CO ratio determines the process in which syngas will be utilized and the type of chemicals it will produce. In the present work, we investigate the effect of H_2_O/CO_2_ (steam/carbon dioxide, S/C) ratio of 0.5, 1 and 2 in the feed, on the electrochemical performance of an 8YSZ electrolyte-supported solid oxide cell and the H_2_/CO ratio in the outlet, under co-electrolysis at 900 °C. The B-site iron doped lanthanum strontium chromite La_0_._75_Sr_0_._25_Cr_0_._9_Fe_0_._1_O_3-δ_ (LSCF) is used as fuel electrode material while as oxygen electrode the state-of-the art LSM perovskite is employed. LSCF is a mixed ionic-electronic conductor (MIEC) operating both under a reducing and oxidizing atmosphere. The cell is electrochemically characterized under co-electrolysis conditions both in the presence and absence of hydrogen in the feed of the steam and carbon dioxide mixtures. The results indicate that under the same concentration of hydrogen and different S/C ratios, the same electrochemical performance with a maximum current density of approximately 400 mA cm^−2^ is observed. However, increasing p(H_2_) in the feed results in higher OCV, smaller iV slope and R_p_ values. Furthermore, the maximum current density obtained from the cell does not seem to be affected by whether H_2_ is present or absent from the fuel electrode feed but has a significant effect on the H_2_/CO ratio in the analyzed outlet stream. Moreover, the H_2_/CO ratio seems to be identical under polarization at different current density values. Remarkably, the performance of the LSCF perovskite fuel electrode is not compromised by the exposure to oxidizing conditions, showcasing that this class of electrocatalysts retains their reactivity in oxidizing, reducing, and humid environments.

## 1. Introduction

Although the COVID-19 pandemic in 2020 caused the largest annual reduction in CO_2_ emissions, we must adapt to a more environmentally friendly lifestyle and rely on low-carbon energy technology. The main sources of greenhouse gas emissions are industry, electricity production, and transportation, which all rely heavily on carbon-based fuels. The environmental threat, along with the depletion of fossil fuel resources and the rapid increase in their costs, have turned the attention of both scientists and policymakers to renewable energy sources (RES). Generating energy from RES (i.e., sun and wind) is of great importance, but when it comes to practice some problems come to light. The energy resulting from such sources exhibits daily, weekly and seasonal variations and therefore, it is quite common to have surplus energy that must be stored or in any case, it will be considered a large loss [1]. At the same time, there are numerous industrial processes in which the heat is considered a waste, while other processes have high heat requirements to operate.

High-temperature (HT) electrolysis was proven to be an effective method of utilizing the abundant CO_2_ in the atmosphere to produce alternative fuels that can replace fossil fuels and at the same time, make efficient use of the available RES [2,3]. The level of carbon dioxide in the atmosphere has exceeded limits (increased to 413 ppm in 2020) [4] and to the benefit of the environment and life itself, its conversion to carbon-based chemicals, such as methane, carbon monoxide, methanol, etc. is highly desirable [4,5,6]. In HT electrolysis, electrical energy is converted into chemical energy, thus allowing excess energy to be stored in chemical form.

A major advantage of solid oxide electrolysis compared to other electrolysis technologies, e.g., PEM or alkaline, is the intrinsic reversibility, which indicates that the same device, i.e., cell or stack, can also operate as a fuel cell to generate electrical energy. Although significant progress was achieved in reversible PEM fuel cells (also known as unitized regenerative PEM fuel cell systems), their roundtrip efficiency is still lower than reversible SOC devices while they require the use of noble metals (Pt, IrO_2_, etc.). Furthermore, the operation of SOCs comes with some advantages, such as the ability to electrolyze not only H_2_O but CO_2_ as well, since both reactions are thermodynamically favored [7,8]. Part of the energy required for electrolysis can be provided as heat under high-temperature electrolysis, rather than at low temperatures, and this allows for lower energy costs for the whole process. With the increase in temperature, the total energy demand (ΔH) is almost constant, while heat demand (T·ΔS) increases and electricity energy demand (ΔG) decreases. In the case of co-electrolysis of steam and CO_2_, it should be considered that H_2_O is thermodynamically more favorable to be electrochemically reduced than CO_2_ since ΔH for the H_2_O dissociation reaction is lower over the entire temperature range.

When renewable or waste energy is being used, syngas and its products (synthetic fuels that are being produced mainly through the Fischer-Tropsch process [7,9,10]) are considered green energy carriers and electrolysis using such energy sources is a ‘power to fuels’ or a ‘power to syngas’ technology. Methane reformation, coal gasification, steam reforming of natural gas, etc., are also syngas production routes [5,11]. In the RES-powered SOEC process, fuels are produced through a carbon-neutral cycle, where CO_2_ is recycled. Furthermore, along with CO and H_2_, high-purity oxygen is also produced and is considered a valuable product [12,13,14].

Due to the high-temperature operation and given the presence of H_2_ in the feed, the reverse water gas shift (RWGS) catalytic reaction (Equation (3)) occurs along with the electrochemical reduction reactions (Equations (1) and (2)). Therefore, syngas is produced either by electrocatalytic, or catalytic reaction, and the origin of carbon dioxide is still disputed [10,15].

(1) $${\mathrm{CO}}_{2}+2e^{-}⇌\mathrm{CO}+O^{2-}\;({\mathrm{CO}}_{2}\;\mathrm{reduction})$$

(2) $$H_{2\;}O+2e^{-}⇌H_{2}+O^{2-}\;(\mathrm{Steam}\;\mathrm{reduction})$$

(3) $${\mathrm{CO}}_{2}+H_{2}⇌\mathrm{CO}+H_{2}O\;(\mathrm{RWGS}\;\mathrm{reaction})$$

Significant advances were made in SOFCs since their emergence [16], while SOECs have followed this development, resulting in similar materials being used in both technologies. The SoA fuel electrode material for SOFCs is the Ni-YSZ or Ni-GDC cermet. However, these materials do not meet the fuel electrode requirements for operation under co-electrolysis conditions [4,7,14,17,18,19]. Considering these, ABO_3_ perovskite oxides have been proposed as alternative electrode materials due to their electrocatalytic activity and high redox stability, such as LaSrCrMnO_3-δ_, LaSrCoMnO_3-δ_, used alone or mixed with electrolyte material (i.e., LSM-YSZ) as both fuel and oxygen electrodes [7,18] depending on the metal ion dopants.

The operating parameters of the co-electrolysis process determine the H_2_/CO ratio in the produced syngas. These parameters include the operating temperature, the fuel electrode material properties, the efficiency, i.e., the current collected by applying overvoltage, and the H_2_O/CO_2_ ratio in the feed. The ratio of H_2_/CO can determine the subsequent Fischer-Tropsch (F-T) process and therefore the type of synthetic fuel that will be produced from it. In most cases, ratios range from 1:1 to 3:1 [10]. For example, from a ratio of 3:1 synthetic natural gas or methane can be formed, while from lower ratios liquid hydrocarbons are produced by the F-T process, while oxo processing or hydroformylation is used to produce aldehydes, which need a ratio of 1:1 [9,18]. In this work, the effect of H_2_O: CO_2_ ratio, 1:2, 1:1 and 2:1, in the electrochemical efficiency of an electrolyte-supported electrolysis cell with doped lanthanum strontium chromite substituted with iron in the B-site, namely La_0_._75_Sr_0_._25_Cr_0_._9_Fe_0_._1_O_3-δ_ (LSCF), as fuel electrode and LSM-YSZ as the oxygen electrode, is investigated under co-electrolysis conditions at 900 °C with and without the co-feed of H_2_ (which acts as a safe gas in the case of SoA electrodes). The production of tailored syngas is a key advantage of solid oxide co-electrolysis and the correlation of the S/C ratio in the feed with it can provide valuable information for the process to be as efficient as possible.

## 2. Materials and Methods

The fuel electrode material La_0_._75_Sr_0_._25_Cr_0_._9_Fe_0_._1_O_3-δ_ (LSCF) perovskite oxide was synthesized using the modified sol-gel method with citric acid, as described in detail in previous work [20,21]. The as-synthesized material in powder form was then ball milled for 48 h with 6–7 mm diameter ZrO_2_ beads to obtain a fine powder, which was then dispersed in a solvent (3% ethyl cellulose in terpineol) to produce an ink with 60% wt solids, which was also ball milled for 48 h.

The fuel electrode, as well as each layer, were screen printed (StV mesh of 40 μm) on the 8YSZ (8% mol Yttria Stabilized Zirconia, Coorstek, Technox 802) type solid disk electrolyte of 20 mm diameter and 1.5 ± 0.5 mm thickness. Between the electrolyte and the electrode, a thin layer of GDC20 (ink 50% wt solids of Gd_0_._20_Ce_0_._80_O_1_._95_, Cerpotech nanopowder in 3% ethyl cellulose in terpineol) was developed by screen printing acting as a barrier layer to avoid interaction of lanthanum strontium chromium ferrite layer (LSCF) and 8YSZ forming a non-conductive LaZr_2_O_7_ layer after prolonged operation at high temperatures. The GDC layer further enhances the increase of TPB and thus its electrocatalytic activity [22,23,24]. On the opposite side of the electrolyte, LSM-YSZ (fuelcellmaterials) was screen printed as the oxygen electrode and LSM (fuelcellmaterials) as the current collector layer. Pt (fuelcellmaterials) paste was used as a fuel electrode current collector. The rationale behind the sequence in which the layers were screen printed in the electrolyte lies in the sintering profile of each. For GDC, LSCF, and LSM-YSZ and LSM the sintering temperatures were 1350 °C (1 h), 1200 °C (3 h), and 1150 °C (2 h), respectively.

The button cell with an active electrode area of ~0.79 cm^2^ was placed in the ProboStat^TM^ (NorECs) set up for the electrochemical characterization, where each side was fed by a defined mixture controlled by electronic mass flow controllers. Steam was fed to the system by passing the reaction mixture through a heated hydrator. By controlling the hydrator temperature, different relative humidity values and thus different H_2_O/CO_2_ (S/C) ratios were achieved. A PGSTAT302N (Metrohm) potentiostat/galvanostat with a frequency response analyzer (FRA32M) was used for the electrochemical measurements and characterization. The cathode outlet stream was analyzed using a non-dispersive infrared analyzer (NDIR) (Fuji, ZRE analyzer), a mass spectrometer (Pfeiffer Vacuum Omnistar, GSD320 MS), and a gas chromatograph (Varian, 3800 GC). The NDIR analyzer offers simultaneous online measurements of up to four different components: CO, CO_2_, CH_4,_ and O_2_. The simultaneous recording of CO, CO_2,_ and H_2_ signals was done by the quadrupole mass spectrometer analyzer with a gas-tight ion source, equipped with Faraday and SEM detectors. Finally, the gas chromatograph, equipped with a thermal conductivity detector and a Haysep-T packed column at 30 °C, was used to quantify the outlet stream. To prevent vapor condensation, cathode inlet line was heated to temperature above 100 °C. A water trap was placed in the outlet cathode line to accumulate water and prevent moisture from entering the analysis system.

After placing it in the reactor, the cell is heated up to 1000 °C with a feed of 5% H_2_ in He and 100% He on the fuel and oxygen side, respectively. At this temperature, the cell performance is studied in fuel cell mode, and then the temperature is reduced to 900 °C with He feeding on both sides. Electrochemical characterization under co-electrolysis conditions for the various S/C ratios included current-potential curves recorded from an open circuit voltage (OCV) to 1.5 V. In addition, AC impedance plots were recorded, and product gas composition was analyzed. Three different S/C ratios were studied utilizing six different mixture compositions:

1. 31% H_2_O-62% CO_2_-4% H_2_ in He (S/C= 0.5)
2. 31% H_2_O-31% CO_2_-19% H_2_ in He (S/C= 1)
3. 62% H_2_O-31% CO_2_-4% H_2_ in He (S/C= 2)
4. 31% H_2_O-62% CO_2_ in He (S/C= 0.5)
5. 31% H_2_O-31% CO_2_ in He (S/C= 1)
6. 62% H_2_O-31% CO_2_ in He (S/C= 2)

On the oxygen electrode side, pure O_2_ is fed in all cases. The total flow is kept equal to 290 mL/min on the fuel electrode side.

## 3. Results and Discussion

### 3.1. Electrochemical Characterization

Polarization curves (iV) were recorded from OCV to 1.5 V (electrolysis mode) and electrochemical impedance spectroscopy (EIS) is performed in the frequency range of 100 kHz−10 mHz with an AC amplitude of 20 mA at OCV and at a fixed current density of 250 mA cm^−2^.

#### 3.1.1. Effect of S/C Ratio with H_2_ Co-Feed in the Fuel Electrode LSCF

**Figure 1:**
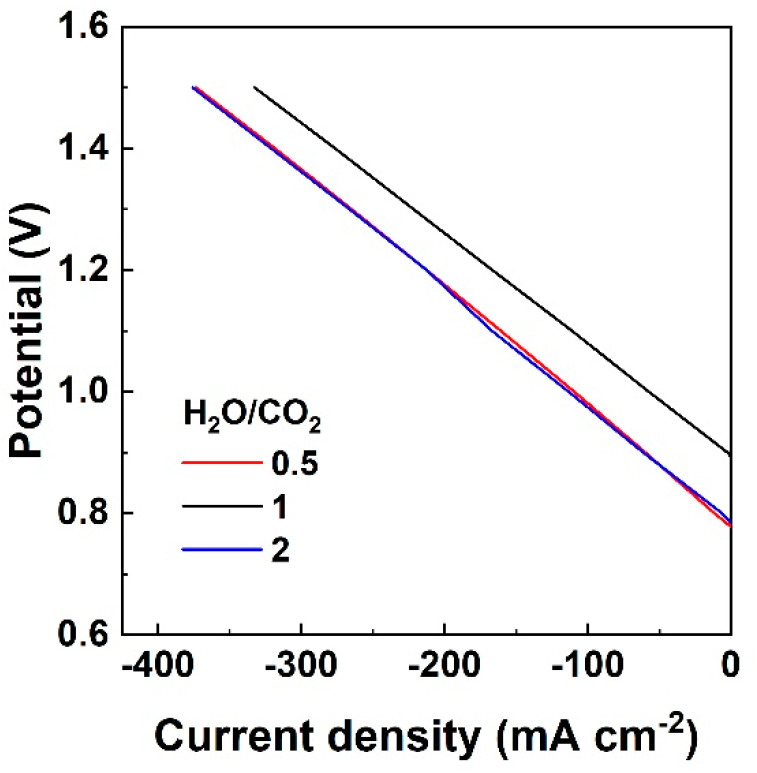
Current density-voltage (iV) curves recorded at 900 °C under electrolysis of CO_2_-H_2_O mixtures, for three S/C ratios of 0.5, 1 and 2, with H_2_ in balance with He in the fuel electrode side and pure oxygen in the oxygen electrode side in all cases.

The characteristic iV curves shown in Figure 1 were recorded under potentiostatic mode for CO_2_-H_2_O-H_2_ gas mixtures corresponding to the three different S/C ratios.

It is noteworthy that in the case of S/C = 1, the open circuit potential is the highest observed. To calculate the thermodynamic Nernst potential, apart from the temperature, the partial pressure of each component is used, which is present on each electrode compartment and participates in the electrochemical reactions. In the case of S/C equal to 1, the H_2_ concentration is higher than for the other two ratios (19% instead of 4%), leading to the observed high open circuit potential difference. The iV curves for the lowest and highest concentration of H_2_O, compared to CO_2_, are almost identical and the maximum current density is approximately 400 mA cm^−2^, while for the equimolar mixture of H_2_O and CO_2,_ the maximum current density is about 350 mA cm^−2^. In this regard, at a constant current density of 250 mA cm^−2^_,_ the overpotential is 0.492, 0.457, and 0.485 V, for S/C of 0.5, 1 and 2, respectively. Lower overpotential indicates lower power consumption and therefore higher efficiency, which based on the above values shows that the S/C = 1 is the most favorable condition. The values of total resistance (ASR) in Ω cm^2^ are calculated from the slopes of the iV curves and are thus equal to 1.936, 1.823, and 1.902 Ω cm^2^ for S/C = 0.5, 1 and 2, respectively. The ASR values appear to increase with a decreasing S/C ratio for a constant H_2_ concentration (S/C of 2 to 0.5), while ASR decreases for a constant steam concentration and higher H_2_ concentration (S/C of 0.5 to 1). When CO_2_ concentration is constant, steam concentration decreases and H_2_ increases, the ASR value decreases (mixtures with S/C of 2 to 1), while OCV increases [25]. The overlap of iV curves for S/C = 0.5 and 2 is an indication that the performance of the cell does not depend on the ratio between steam and carbon dioxide. This was reported in the relevant literature [26] for other fuel electrode materials, such as the SoA Ni-YSZ. The reason is that under these conditions, the rates for H_2_O and CO_2_ electrolysis are similar. This is depicted by the estimated ASR values which are similar for both S/C ratios, i.e., only a small decrease in ASR is observed when the concentrations of CO_2_ and H_2_O are reversed.

The perovskite-type mixed ionic-electronic conductor used as the fuel electrode in this study does not need to be in its reduced form to be a functional cathode, unlike the Ni-based SoA materials. However, to have a direct comparison with the state-of-the-art nickel-based materials and to examine how the catalytic reverse water gas shift reaction affects the performance and the reaction pathways, hydrogen is co-fed to the cathode. In a recent work [26], where different S/C ratios were studied with SoA Ni-GDC as the fuel electrode at 900 °C, similar performance to the cell studied in the present work was reported. It should be noted that in present work, the electrolyte used is much thicker (1.5 mm) compared to the electrolyte used in [26] (150 μm), indicating the higher electrocatalytic activity of LSCF compared to Ni-GDC. Hydrogen does not participate in the electrochemical reduction processes under the electrolysis mode, but only in the catalytic RWGS reaction to produce CO and H_2_O, with the latter competing with the electrochemical decomposition of CO_2_ at the TPB sites [25]. Under open circuit conditions, the only process taking place at the cathode side is the endothermic catalytic RWGS reaction (Equation (3)), and the composition of the mixture is defined assuming thermodynamic equilibrium. The equilibrium compositions for the different inlet feeds were calculated using the corresponding thermodynamic data and gas properties in MATLAB platform and are given in Table 1. Other possible reactions occurring under highly reducing conditions (e.g., the formation of carbon or methane) were not considered in the thermodynamic analysis, as there was no experimental indication of either coke or methane formation. When a current or potential is applied to the cell, the electrochemical reductions of CO_2_ (Equation (1)) and/or H_2_O (Equation (2)) are also initiated at the electrochemically active sites of the cathode. Therefore, two parallel reactions occur with regard to CO_2_ conversion: the catalytic RWGS process (Equation (3)) by the effect of the hydrogen fed to the cell or produced in-situ by the electrolysis reaction (Equation (1)), and the direct electrochemical reduction of CO_2_. Similarly, regarding H_2_O, it is produced through the RWGS reaction (Equation (3)) and consumed through its direct electrochemical reduction (Equation (2)).

The Nernst potential under co-electrolysis is calculated using the partial pressures of the gaseous species: cathode CO_2_-CO or H_2_O-H_2_/anode O_2_, while p(O_2_) is equal to 1 in each case. The equilibrium mixture composition resulting from feeding the different mixtures is used to calculate the theoretical OCV and the values are given in Table 1. These calculations confirm the experimental observation of a higher OCV for the mixture where steam and carbon dioxide are equally fed to the cathode and the higher H_2_ concentration. The experimental OCV values are close to those calculated using p(H_2_O) and p(H_2_) and to those using p(CO_2_) and p(CO). Any deviation between the experimental OCV and the theoretical Nernst potential values can be due to either an electronic leak (eddy current) or a difference in gas composition in case of poor sealing between the anode and cathode chambers [27]. However, if this is the case, it applies to all measurements conducted under the same conditions and the qualitative trend still holds.

**Figure 2:**
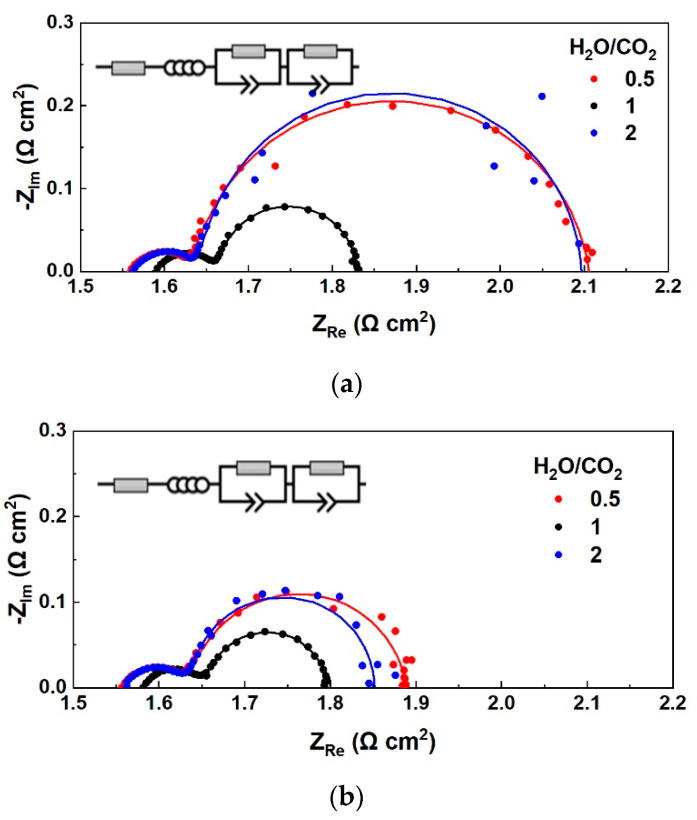
The electrochemical performance of the button cell under co-electrolysis. Nyquist plots recorded at (**a**) 0 mA cm^−2^ (open circuit) and (**b**) 250 mA cm^−2^ for different H_2_O/CO_2_ ratios in the fuel electrode feed with H_2_ co-feed, balanced with He, for a frequency range of 100 kHz−10 mHz and an amplitude of 20 mA.

The ASR values calculated from the slope of iV curves correspond to the total resistance of the cell, i.e., the sum of the ohmic and the polarization resistance. However, DC characterization (iV curves) cannot distinguish the individual contribution of each resistance, and therefore AC characterization using electrochemical impedance spectroscopy is required to analyze a practical electrochemical cell. The recorded Nyquist plots at OCV for the three different CO_2_-H_2_O-H_2_ mixtures at 900 °C are shown in Figure 2a. The amplitude under which the plots were recorded in OCV, i.e., the zero current, from high to low frequency, is ±20 mA, which indicates the recorded AC gives information on the losses of the cell in fuel cell and electrolysis cell operation, due to its ability to operate in the reversible oxidation-reduction mode [11]. The fitted data shown as lines together with the EIS data obtained in real-time (shown as dots), were collected by fitting the EIS data using Relaxis impedance analysis software and the equivalent circuit R_s_L-(R_1_CPE_1_)(R_2_CPE_2_) as shown in the AC figures. The circuit consists of an electrolyte resistor, an inductor, and two circuits representing the two electrodes, connected in series.

Under different conditions, such as pressure, temperature, and feed gas composition, the resistances of a cell change, and this is reflected in the AC plots. For S/C = 2, the intercept of the impedance plot at high frequency, corresponding to the ohmic resistance, R_s_, is almost the same as the R_s_ value when S/C = 0.5, both in open-circuit conditions and current load. The polarization resistance (R_p_) is calculated as the difference of the intercept of the impedance plot at low frequency minus the R_s_. The high-frequency arc, which is associated with the activation and concentration resistances of the electrodes, as well as the low-frequency arc, which is consistent with the diffusion resistances, are the same for these two different S/C ratios mentioned, under open circuit conditions (Figure 2a). This indicates that not only R_s_ is unaffected by the different S/C ratios, but also kinetics remains the same if steam or CO_2_ is in excess in the fuel electrode feed, at a low H_2_ concentration (4%).

In the case where the H_2_ concentration is high (19%), while steam and CO_2_ are fed equally to the cathode (S/C = 1), the series resistance, R_s,_ is slightly higher, while the polarization resistance, R_p_, and the total resistance are much lower than for S/C = 0.5 or 2. In other words, the high-frequency arcs of the impedance spectra in Figure 2a are almost the same, while there is a deviation in the low-frequency arcs, which indicates the ease of diffusion of the light H_2_ molecules since in η_conc_ (and for R_p_) the microstructure parameters are essential [28,29]. When S/C in the feed is 1, the equilibrium S/C composition is calculated to be 1.5. The higher H_2_ concentration in the feed leads to a decrease in R_p_ and, to a lesser extent, in the total resistance, while accounting for more CO production, through the RWGS reaction. [28,29]. The conductivity of LSCF appears to be lower (higher R_s_) at high H_2_ concentration, in agreement with literature results [30].

**Figure 3:**
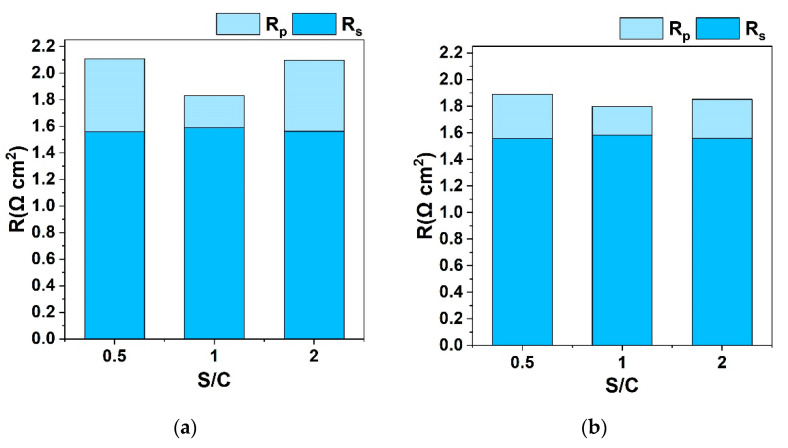
Ohmic (R_s_) and polarization (R_p_) resistance values obtained from fitting EIS data from Figure 2a,b that recorded under co-electrolysis at (**a**) 0 mA cm^−2^ (open circuit) and (**b**) 250 mA cm^−2^ for different H_2_O/CO_2_ ratios in the fuel electrode feed with H_2_ co-feed.

The impedance spectra recorded at a constant current density of 250 mA cm^−2^ are given in Figure 3b. A small increase in R_s_ and a decrease in R_p_ values is observed compared to the impedance spectra in OCV (Figure 2a). The ASR values calculated from the slopes of the iV curves in the range from OCV to 1.5 V, match with the total resistance of the EIS plots, with a deviation of 1.6–1.8%. The R_p_ values for S/C = 2 and 0.5 are similar, which could indicate that CO_2_ is also involved in the electrochemical reactions taking place at the fuel electrode [11]. Nyquist plots recorded in OCV but also under constant load for high H_2_ concentration and S/C = 1 have smaller high-frequency arcs, representing the electrochemical reaction process [25].

The ohmic and polarization resistances obtained by fitting EIS data from Figure 2 are given in Figure 3. When H_2_O is equal to CO_2_ in the feed, i.e., S/C = 1, the total resistance is lower compared to the other two cases where S/C is 0.5 and 2, which indicates faster kinetics for the equimolar reaction mixture [18]. Impedance plots under current load show a similar performance; polarization resistances decrease for each S/C ratio, indicating enhanced kinetics [25,31]. For the different S/C ratios studied, a significant change is observed in the charge transfer resistance, R_p_, of the cell, while the series resistance, R_s_, remains almost unaffected. This increase of R_p_ for the different S/C ratios is less pronounced under electrolysis mode (250 mA cm^−2^).

#### 3.1.2. Effect of S/C Ratio without the Co-Feed of a Reducing Agent on the Fuel Electrode LSCF

**Figure 4:**
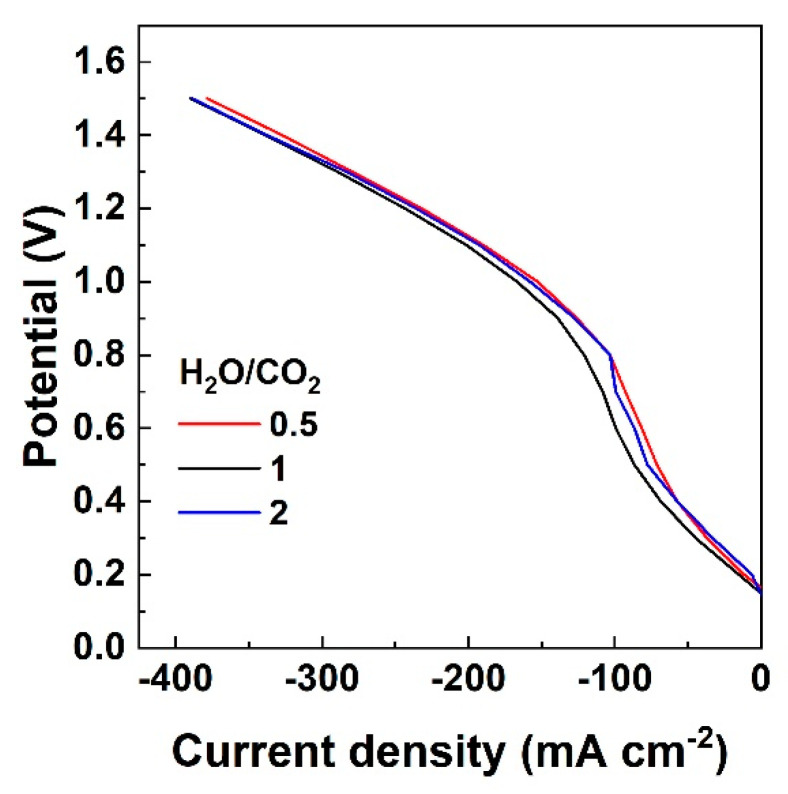
Current density-voltage (iV) curves recorded at 900 °C under electrolysis of the H_2_O/CO_2_ mixtures, for three S/C ratios of 0.5, 1 and 2, in balance with He in the fuel electrode side and pure oxygen in the oxygen electrode side in all cases.

The electrochemical performance of the cell is also studied with CO_2_ and H_2_O feeding, without H_2_ co-fed on the cathode side. The OCV in this case, where the reducing gas H_2_ is absent, is approximately 0.2 V which agrees with other studies reported in the literature [18,32]. The iV curves recorded in the absence of H_2_ in the fuel inlet show a significant difference from the case where H_2_ is co-fed. Namely, the iV curves from OCV to 1.5 V follow a non-linear evolution since the Nernst potential is not constant and a significant concentration of H_2_ is required to be produced for the iV curve to follow the linear relation. The polarization curves become linear at higher current density, higher than 100 mA cm^−2^ for an S/C 0.5 and 2 and higher than 150 mA cm^−2^ for an S/C of 1, as shown in Figure 4, while at lower current values the cell potential rises abruptly. In this region, where the current density values are low, electrolysis occurs to produce H_2_ and/or CO, and the non-linear behavior of the polarization curves [31] changes when the cell potential reaches the electromotive force and, in the meantime, the reference potential is reached.

When H_2_ is co-fed with the CO_2_ and H_2_O reaction mixture, the iV curves exhibit the typical, linear trend over the entire current density range. Under the different H_2_O/CO_2_ ratios, the electrochemical performance does not show significant differences, in agreement with other relevant work [18], with the maximum current density being a little lower for the lowest H_2_O concentration, while for the other two steam contents, the highest current density is recorded. Furthermore, the current density at 1.5 V is similar to the case where H_2_ is co-fed to the cathode, which is a strong advantage of perovskite materials over Ni-based materials, as they can operate efficiently under extreme conditions.

**Figure 5:**
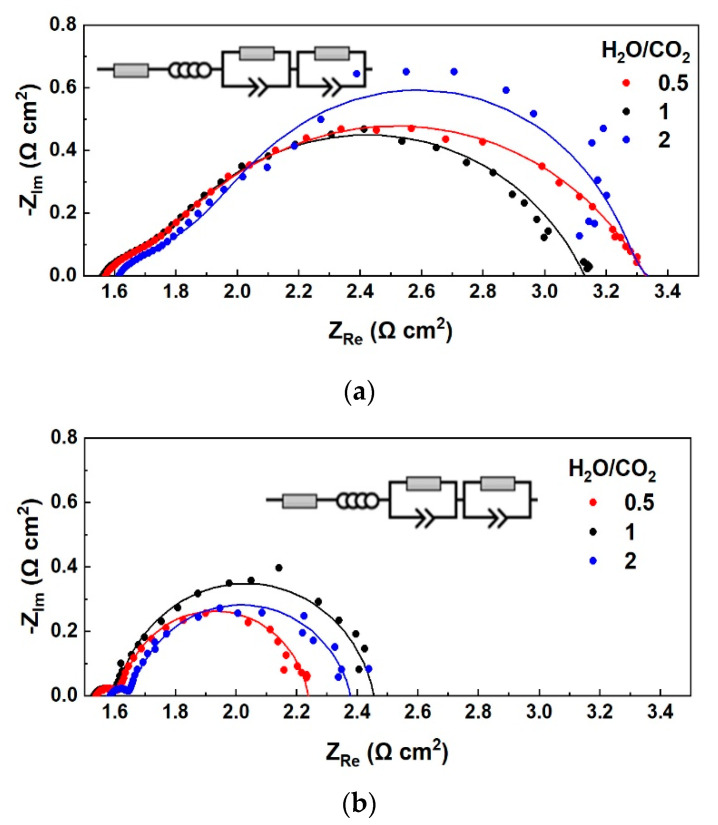
The electrochemical performance of the button cell under co-electrolysis. Nyquist plots recorded at (**a**) 0 mA cm^−2^ (open circuit) and (**b**) 250 mA cm^−2^ for different H_2_O/CO_2_ ratios in the fuel electrode feed in balance with He for a frequency range of 100 kHz–10 mHz and an amplitude of 20 mA.

AC plots recorded under OCV and 250 mA cm^−2^ when only CO_2_ and H_2_O are fed in the cathode side at 900 °C are shown in Figure 5. The polarization resistance does not seem to be affected by the different H_2_O concentrations, while the total resistance decreases for S/C from 0.5 to 2, to 1, as shown in Figure 5a.

**Figure 6:**
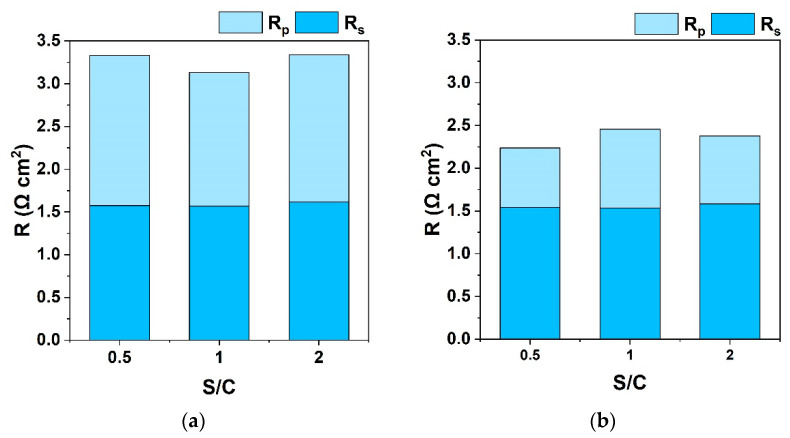
Ohmic (R_s_) and polarization (R_p_) resistance values obtained from fitting EIS data from Figure 5a,b that recorded under co-electrolysis at (**a**) 0 mA cm^−2^ (open circuit) and (**b**) 250 mA cm^−2^ for different H_2_O/CO_2_ ratios in the fuel electrode feed in balance with He.

The polarization resistance decreases significantly under load, indicating that diffusion of reactive species into the active sites is faster under bias compared to that under OCV [18]. Comparing the different impedance plots in Figure 5b, the lower R_p_ corresponds to the mixture where the CO_2_ concentration is twice the H_2_O concentration, as shown by the fitted R values in Figure 6. The higher R_p_ is observed for the equimolar H_2_O and CO_2_ feed, as a result of their competitive adsorption/desorption, diffusion, and electrochemical reduction at the TPB sites.

An analysis of the resistances derived from Nyquist plots reveals a difference in the interpretation between the two cases studied: presence and absence of hydrogen. Specifically, in the presence of hydrogen, the R_p_ values follow the same order as H_2_ concentration in the reaction mixture (13%, 2.8% and 1.6% for S/C = 1, 2 and 0.5, respectively). This can be attributed to the lower diffusion resistance of the low molar weight H_2_ molecule, since in concentration overpotential (and therefore for the low frequency arc of R_p_) the microstructure parameters are essential [28,29]. In the case of absence of H_2_ in the gas feed (a small amount of hydrogen is only produced electrochemically), the R_p_ presents a reverse trend, with the higher R_p_ observed for S/C = 1 (equimolar H_2_O and CO_2_ feed), as a result of their competitive adsorption/desorption, diffusion, and electrochemical reduction at the TPB sites. Therefore, in the latter case, it is the gas reactants that define R_p_ while H_2_ diffusion is the dominant factor in the former case. In any case, it should be highlighted that these changes in resistances are not very prominent, given the wide range of S/C ratios studied, and the total electrochemical performance of the cell is not remarkably affected by gas composition, as depicted in Figure 1 and Figure 4.

### 3.2. Outlet Gas Analysis

The outlet stream of the cathodic compartment is quantitatively analyzed by gas chromatography (GC) after removing water through a trap to protect the analysis system. The exhaust gas composition is studied with varying key operational parameters, i.e., load and S/C ratio, at the cathode inlet.

Under OCV, the RWGS defines the gas mixture given that H_2_ is co-fed to the cathode inlet until equilibrium is reached. The equilibrium cell voltage is calculated using the partial pressure of components, p(CO_2_), p(CO), p(H_2_O), and p(H_2_), using the obtained GC data (OCV_GC_), and the thermodynamically expected values (OCV_exp_). Both values are given in Table 2 where a slight difference between these values is noticed.

**Figure 7:**
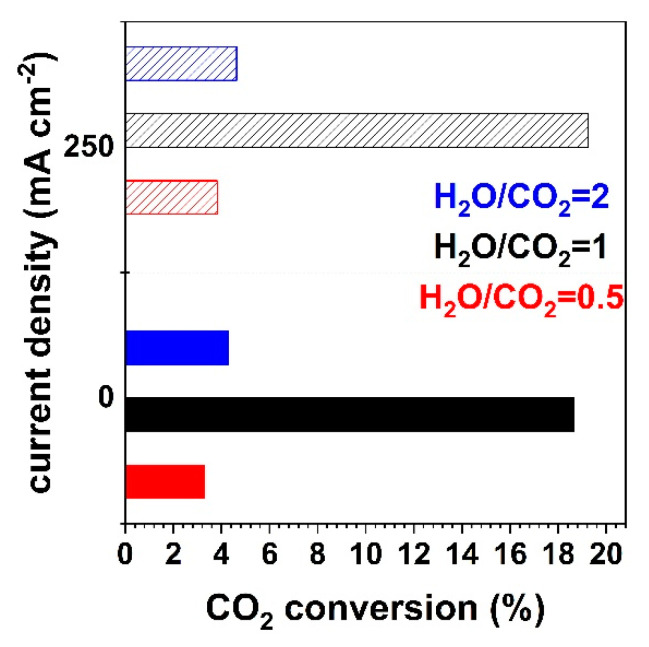
Conversion of CO_2_ under a current density of 0 mA cm^−2^ (open circuit) and 250 mA cm^−2^ for different H_2_O/CO_2_ ratios of 0.5, 1 and 2, with H_2_ co-feed in the cathode, at 900 °C.

The CO_2_ conversion values at 900 °C for the three different cathode feed mixtures under OCV, i.e., when only the RWGS reaction takes place, and under galvanostatic operation, i.e., when both RWGS reaction and electrolysis take place, are shown in Figure 7. Under OCV, CO_2_ conversion is significantly affected by the H_2_ concentration in the feed, process temperature, electrode catalytic properties, etc., as CO is only produced through the catalytic RWGS reaction. As a result, the CO_2_ conversion is higher when the H_2_ concentration is higher, and these values agree with the literature [10]. This is also observed under the current application, but with a small increase in CO_2_ conversion, for all S/C ratios tested due to low current and therefore low Faradaic conversion of the reactants. By holding constant the H_2_ and the fuel concentration (steam plus carbon dioxide) in the cathode feed, the CO_2_ conversion is higher when the steam concentration is higher.

It should be noted that the low CO_2_ conversion values reported are typical for a differential reactor employing a small area button cell, as in the present study. Furthermore, as it generally occurs with button cells, the total gas flow (290 mL/min) was significantly larger than that corresponding to the stoichiometry of the faradaic reaction. The total CO_2_ conversion is low for practical applications; however, the present analysis of the effect of operating conditions (mainly the S/C ratio) contributes to a better understanding of the reaction mechanism combining catalytic and chemical processes. In any case, the CO_2_ conversions are similar to those reported for Ni-YSZ electrodes in button cell configuration under similar conditions [33].

**Figure 8:**
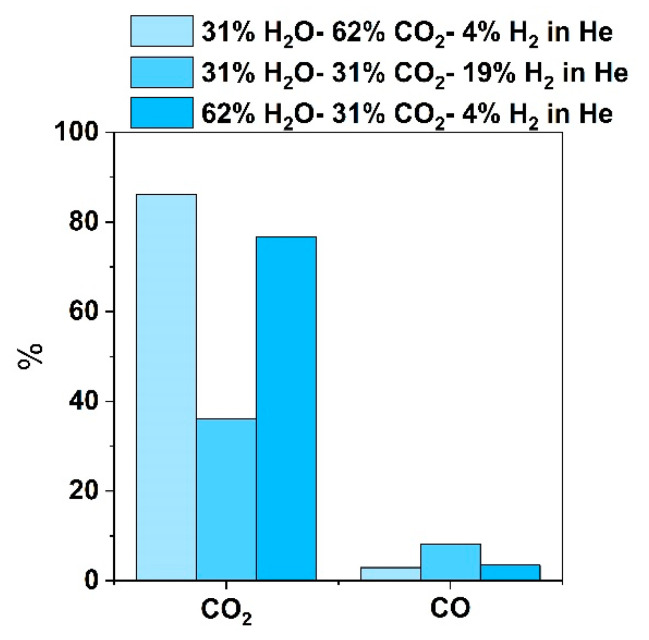
Outlet composition of CO_2_ and CO in the outlet steam (dry basis analysis) under the galvanostatic operation of the button cell at a current density of 250 mA cm^−2^ for the three different cathode feed mixtures with an increasing S/C ratio from 0.5 to 2, at 900 °C.

The dry basis CO_2_ and CO composition in the outlet cathode stream is given in Figure 8, for the same current density of 250 mA cm^−2^ as the CO_2_ conversion shown in Figure 7. The results are presented on a dry basis since before cathode outlet analysis, the steam is condensed and removed as liquid water through a water trap. The CO_2_ concentration in the feed on a dry basis is 89, 44, and 80% for S/C is equal to 0.5, 1 and 2, respectively.

The electrochemical CO_2_ reaction pathway parallels steam electrolysis and in the presence of significant H_2_ content is not elucidated, due to enhanced kinetics and since CO_2_ is also less favorable to be electrochemically reduced when H_2_O is present. Therefore, it is not clear whether CO production originates from the CO_2_ electrochemical decomposition or through the RWGS reaction. There have been different approaches in the literature to further investigate this. According to Faro et al. [33,34], the ratio of the total CO produced to H_2_ in the inlet (ξ_CO,chem_) or CO produced electrochemically (I/2F) (ξ_CO,Faradaic_) can indicate whether CO is produced solely from the RWGS reaction or if electrolysis takes place along with it. If ξ_CO,chem_ is higher than 1, this indicates that CO is also produced via electrolysis reaction, since the H_2_ conversion comes only from the RWGS reaction taking place in an open circuit. If ξ_CO,Faradaic_ is higher than 1, the electrochemical and the catalytic processes contribute to CO production. The ξ_CO,chem_ value is larger when the H_2_ concentration in the feed is low, and closer to 1 when H_2_O is half the CO_2_ concentration in the feed, and in all cases ξ_CO,chem_ is lower than 1, so CO_2_ electrolysis is not that possible. The ratio of CO produced to the moles produced electrochemically by applying a current density of 250 mA cm^−2^ (ξ_CO,Faradaic_), is greater than 1 and this strengthens the assumption that CO production can be attributed mainly to the RWGS reaction. On the other hand, when H_2_ is absent from the feed, only ξ_CO,Faradaic_ can be calculated and it is lower than 1 and indicates that CO_2_ electrolysis at 250 mA cm^−2^ under the H_2_O/CO_2_ feed and different S/C ratios is likely to occur with the catalytic reaction increasing the H_2_O content in the feed decreases ξ_CO,Faradaic_ as well.

**Figure 9:**
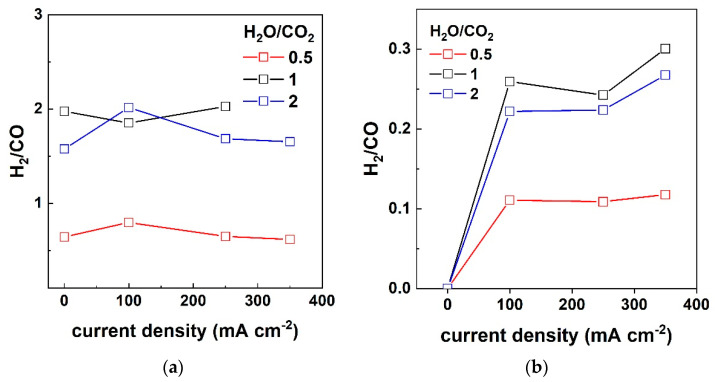
The ratio of H_2_/CO, as calculated from the outlet analysis, under open circuit and electrolysis conditions (100–350 mA cm^−2^) for three S/C ratios: 0.5, 1 and 2 when H_2_ is (**a**) present and (**b**) absent from the feed, at 900 °C.

The H_2_/CO ratio in the outlet cathode stream for both cases (H_2_ presence or absence in the feed) is given in Figure 9. When H_2_ is fed to the cathode, the ratio of H_2_/CO in the produced syngas is defined by the S/C inlet ratio rather than the applied current [8]. For S/C = 1 and 2 ratios, synthesis gas is produced with a similar composition, i.e., an H_2_/CO ratio of 1.5–2, while when the inlet has an S/C = 0.5 the resulting syngas has a composition of H_2_/CO 0.5–0.8 [10].

A similar study also observed an increase in the H_2_/CO ratio with an increasing S/C ratio in the feed [10]. The effect of current is pronounced in the case where H_2_ is absent from the feed (Figure 9b) since H_2_/CO rises abruptly when electrolysis takes place, i.e., under the current application. As in the previous case, the resulting syngas compositions for S/C 1 and 2 are similar, while the low S/C of 0.5 results in a lower H_2_/CO ratio. For current densities of 100 and 250 mA cm^−2^, the H_2_/CO ratio appears to be constant, while a slight increase in H_2_/CO produced is observed at 350 mA cm^−2^.

## 4. Conclusions

Fuel flexibility is a major advantage of solid oxide electrolysis technology; in this way, various renewable fuels can be recycled. The composition of these feedstocks comes from the production process, while no additional processing steps are needed and they are used as such, regardless of the concentration of reactants. In this paper, we studied the electrochemical performance of button cells under co-electrolysis for various S/C ratios in the fuel electrode side feed mixtures. Regarding the effect of the S/C ratio on the composition of the produced syngas, the H_2_/CO ratio was found to be higher when S/C is equal to 1 and 2 compared to a low S/C ratio of 0.5. This trend is observed whether H_2_ was co-fed to the cathode or not. In the absence of H_2_ in the gas inlet, the application of current, i.e., the onset of electrolysis, is essential to kick-start the nexus of reactions and products. However, increasing the current density from 100 to 350 mA cm^−2^ had a marginal effect on the H_2_/CO ratio. An important finding of this study is that the electrochemical performance is not affected by the steam-to-carbon dioxide ratio. EIS was applied to further understand the electrode processes and revealed that in presence of hydrogen in the reaction mixture, hydrogen diffusion defined polarization resistances, while in absence of hydrogen, the competitive adsorption/desorption, diffusion of steam and carbon dioxide are the dominant factors. In addition, the cell performance was not compromised by the absence of H_2_ in the fuel electrode feed, revealing the high stability of LSCF in both reducing and oxidizing atmospheres.

## Figures and Tables

**Table 1:** Inlet feed composition and thermodynamic equilibrium gas composition for each saturated feed mixture studied and OCV calculated using the equilibrium compositions applied for p(CO_2_-CO) and p(H_2_O-H_2_), at 900 °C.

Inlet Feed H_2_O/CO_2_ Ratio	Equilibrium Gas Composition ^1,2^	OCV_theo/CO2-CO_ (mV)	OCV_theo/H2O-H2_ (mV)	OCV_exp_ (mV)
0.5	33.0%H_2_O- 59.4% CO_2_- 1.6% H_2_- 2.2% CO	779	778	773
1	37.2%H_2_O- 24.2% CO_2_- 12.8% H_2_- 6.5% CO	885	884	893
2	62.6%H_2_O- 29.8% CO_2_- 2.8% H_2_- 1.0% CO	783	782	782

^1^ All mixtures are in balance with He gas flow. ^2^ Thermodynamic equations applied in MATLAB.

**Table 2:** Experimental OCV and calculated Nernst potential using both thermodynamic equilibrium gas compositions and experimental gas compositions for p(_CO2-CO_) and p(_H2O-H2_) at open circuit voltage, at 900 °C.

Inlet Feed Composition ^1^	OCV_exp_ (mV)	OCV_GC-CO2/CO_ (mV)	OCV_GC-H2O/H2_ (mV)
31% H_2_O-62% CO_2_-4% H_2_ (S/C = 0.5)	773	765	784
31% H_2_O-31% CO_2_-19% H_2_ (S/C = 1)	893	862	894
62% H_2_O-31% CO_2_-4% H_2_ (S/C = 2)	782	779	775

^1^ All mixtures are in He balance.

## Data Availability

Not applicable.
